# Warming puts the squeeze on photosynthesis – lessons from tropical trees

**DOI:** 10.1093/jxb/erx114

**Published:** 2017-05-30

**Authors:** Mirindi Eric Dusenge, Danielle A. Way

**Affiliations:** 1 Department of Biology, University of Western Ontario, London, ON N6A 5B7, Canada; 2 Nicholas School of the Environment, Duke University, Durham, NC 27708, USA

**Keywords:** Carbon uptake, climate change, dark respiration, global warming, photosynthetic acclimation, temperature-response curve, thermal acclimation, tropical forest.

## Abstract

This article comments on:

Slot M, Winter K. 2017. Photosynthetic acclimation to warming in tropical forest tree seedlings. Journal of Experimental Botany 68, 2275–2284.

**Tropical forests are regions of relative thermal stability and so, although equatorial regions are expected to experience less climate warming than the global average in coming years, tropical trees might be more vulnerable to change. But are they? In this issue of *Journal of Experimental Botany*, Slot and Winter (2017) provide one of the most comprehensive studies on thermal acclimation of tropical trees to date.**

Climate change will increase global temperatures by 2–4 °C in the next 85 years. While this represents an enormous shift in the Earth’s climate, warming is not expected to be uniform over the globe, with equatorial regions warming by ‘only’ 1–2 °C by 2050 (IPCC, 2013). This might lead to the conclusion that tropical forests are therefore less at risk from climate warming than other biomes (Sala *et al.*, 2000). However, tropical forests are regions of thermal stability: on a geological timescale, they have avoided the repeated glaciations and associated climate extremes experienced by higher latitudes. On much shorter timescales, diurnal temperatures may fluctuate by only 5 °C, while monthly mean temperatures may differ by just 1–4 °C across the year (Trewin, 2014), an enormous contrast to the broad temperature swings that temperate and boreal trees experience on a daily and yearly basis.

It has thus long been thought that tropical species may be adapted to a narrow thermal niche and that the ability to tolerate and acclimate to temperatures outside this temperature range may be much more limited than it is in higher latitude species (Janzen, 1967). If this is true, then the relatively small increases in temperatures expected in low latitudes may actually cause greater thermal stress in tropical forests than the larger degree of warming will in temperate and tropical trees. Indeed, increased growth temperatures decrease tree growth in tropical species in almost every study (Way and Oren, 2010). Given that tropical forests contain more than 50% of the carbon found in forests (Pan *et al.*, 2011) and that the majority of the world’s biodiversity is in the tropics (Lewis, 2006), declines in the growth, carbon sequestration and survival of tropical tree species in a warmer world would have dire consequences.

## Thermal acclimation capacity of tropical tree species

While we have considerable data on how temperate species respond to increased growth temperatures, there are only a handful of studies looking at the thermal acclimation capacity of tropical tree species, and this paucity of information impedes our ability to predict how low-latitude forests will respond to a future, warmer world. The new paper by Slot and Winter (2017) provides one of the most comprehensive studies on thermal acclimation of tropical trees to date. They grew seedlings of three common lowland tropical species at 25 °C, 30 °C and 35 °C and assessed how photosynthesis, respiration and growth were affected by the different temperature regimes.

The good news is that all the species acclimated to the warmer temperatures: the thermal optimum of photosynthesis (*T*_opt_, the temperature at which carbon uptake is maximized) increased with increasing growth temperature, and respiration rates were lower in plants from warmer treatments (indicating a reduction in carbon losses). But there was also bad news. The shift in *T*_opt_ was smaller than the shift in growth temperature, net photosynthetic rates at the growth temperature (*P*_growth_, the most ecologically relevant measurement of CO_2_ uptake) were reduced in plants grown at the warmest temperature, and the photosynthetic capacity of leaves showed little plasticity to growth temperature. Most strikingly, one of the three species (*Calophyllum longifolium*) grew so poorly at 35 °C that Slot and Winter had to use a 33 °C treatment to provide enough leaves to collect their data. Even under this lower, ‘severe’ warming treatment, the late-successional *C. longifolium* showed substantial reductions in photosynthesis compared to seedlings grown at 25 and 30 °C, and also compared to the other species in the study, *Ficus insipida* and *Ochroma pyramidale*, which are both early-successional. Overall, the results indicate that while photosynthesis in the study species shows some plasticity to increasing temperatures, acclimation cannot keep pace with warming, and this failure to acclimate successfully may be worse in late-successional species, as also seen in Cheesman and Winter (2013).

## **High-temperature CO**_**2**_ **compensation point**

One of the most interesting parts of the work by Slot and Winter (2017) was their assessment of the high-temperature CO_2_ compensation point, the upper leaf temperature at which net CO_2_ assimilation rates were zero (*T*_max_; see Box 1). Recent work has explored how thermal acclimation affects photosynthetic traits such as *T*_opt_ and *P*_growth_, (Way and Yamori, 2014; Yamori *et al.*, 2014). Also, Yamori *et al.* (2014) noted that the span of leaf temperatures that realizes 80% of the maximum photosynthetic rate was invariant with growth temperature, implying that the temperature response of net photosynthesis is not narrowed or broadened by warming. However, there is almost nothing known about how *T*_max_ is affected by changes in growth temperature. In their study, Slot and Winter (2017) found that a 10 °C change in growth temperature had no effect on *T*_max_, but *T*_max_ did vary between species: while *T*_max_ was 45 °C in *C. longifolium* (the late-successional species with pronounced mortality at 35 °C), *T*_max_ was 50 °C for both *F. insipida* and *O. pyramidale*. The combination of a shift in *T*_opt_ without a corresponding shift in *T*_max_ in plants grown at warmer temperatures resulted in a narrowing of the temperature-response curve of photosynthesis.

**Box 1. Temperature response of net photosynthesis to increasing growth temperature**

The solid, blue line represents a cool-grown leaf and the dashed, red line represents a warm-grown leaf. Plants grown at higher temperatures usually exhibit an increased photosynthetic thermal optimum (*T*_opt_, shown as a point on each curve), but there is little data on how *T*_max_ (the upper temperature at which net CO_2_ assimilation rates are zero, i.e. carbon gain balances carbon loss) responds to warming. If *T*_opt_ increases but *T*_max_ remains constant, as in Slot and Winter (2017), the temperature response of net photosynthesis is ‘squeezed’ and becomes narrower.

**Figure:**
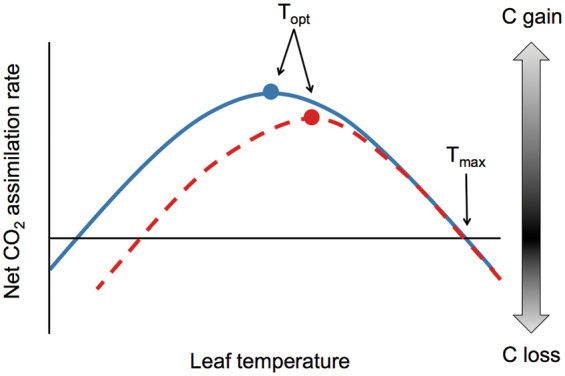

To further explore the extent to which *T*_max_ changes in response to an increase in growth temperature, we collated data from 34 published studies (Box 2; Table 1) that reported temperature-response curves of net photosynthesis for plants grown at two or more different thermal regimes. Only papers with measurements that included points of declining net CO_2_ assimilation rates above the *T*_opt_ were used, ensuring a robust estimate of *T*_max_. We then estimated *T*_max_ for both control and warm-grown plants for each reported species using a second-order polynomial fit to the temperature-response curve of net photosynthesis. Although there is considerable variation in the relationship between the degree of warming and the shift in *T*_max_, overall, a 1 °C increase in growth temperature led to a 0.4 °C increase in *T*_max_. Unfortunately, there is insufficient data to determine if there are significant differences in the thermal acclimation of *T*_max_ between plant functional types, but in 25% of the cases assessed, *T*_max_ actually *decreased* with increasing growth temperature (Box 2). Based on these findings, the inability of the tropical species investigated in Slot and Winter (2017) to shift their *T*_max_ is uncommon, and may be related to the high values for *T*_max_, which are close to temperatures that can cause irreversible damage to leaves (Krause *et al.*, 2010; 2015).

**Box 2. Increasing growth temperatures alter the high-temperature CO_2_ compensation point**

Change in *T*_max_ (∆ T_max_) of net CO_2_ assimilation rate as a function of the increase in growth temperature (∆ T_growth_) in plant species from four plant functional types (see key). Each point plotted represents a comparison between cool and warm-grown plants from a single study (Table 1). The dotted line shows the regression for all data taken together (*y*=–1.29 + 0.40*x*; *r*^2^=0.13; *P*=0.0002).

**Figure:**
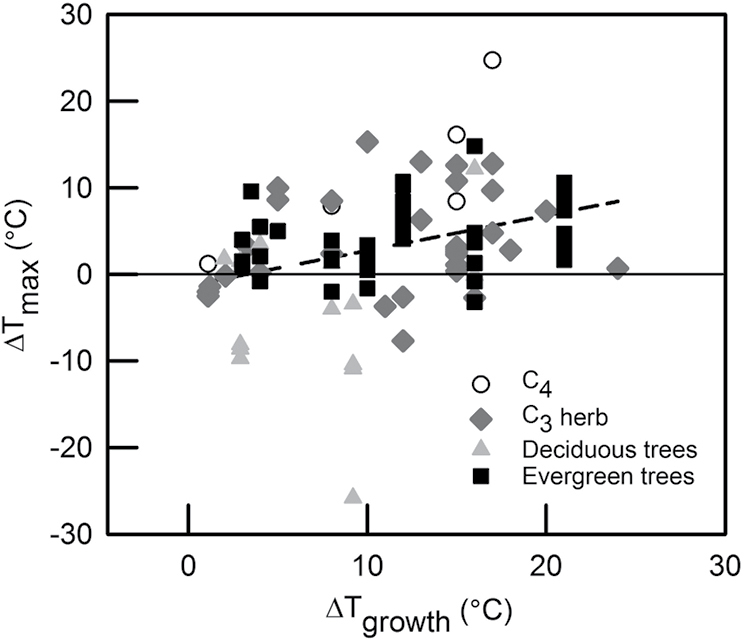

**Table 1.** *Species/functional types used in the high-temperature CO*_*2*_ *compensation point analysis*

**Species**	**Functional type**	**Source**
*Agropyron smithii*	C_3_ herb	Monson *et al.*, 1983
*Albutilon theophrasti*	C_3_ herb	Ziska, 2001
*Amaranthus retroflexis*	C_4_	Pearcy *et al.*, 1981
*Ambrosia psilostachya*	C_3_ herb	Zhou *et al.*, 2007
*Aster ericodes*	C_3_ herb	Zhou *et al.*, 2007
*Atherosperma moschatum* F700	Evergreen tree	Read and Busby, 1990
*Athrotaxis selaginoides* F980	Evergreen tree	Read and Busby, 1990
*Betula payrifera*	Deciduous tree	Dillaway and Kruger, 2010
*Bouteloua gracilis*	C_4_	Monson *et al.*, 1983
*Buchloe dactyloides*	C_4_	Monson *et al.*, 1983
*Calamagrostis canadensis*	C_3_ herb	Kubien and Sage, 2004
*Carex eleocharis*	C_3_ herb	Monson *et al.*, 1983
*Carex eleocharis*	C_3_ herb	Veres and Williams III, 1984
*Chenopodium album*	C_3_ herb	Pearcy *et al.*, 1981
*Colobanthus quitensis*	C_3_ herb	Xiong *et al.*, 2000
*Deschampia antarctica*	C_3_ herb	Xiong *et al.*, 2000
*Dicoria canescens*	C_3_ herb	Toft and Pearcy, 1982
*Eucalyptus camaldulensis* (Qld)	Evergreen tree	Ferrar *et al.*, 1989
*Eucalyptus camaldulensis* (Vic)	Evergreen tree	Ferrar *et al.*, 1989
*Eucalyptus globulus*	Evergreen tree	Crous *et al.*, 2013
*Eucalyptus incrassata*	Evergreen tree	Ferrar *et al.*, 1989
*Eucalyptus miniata*	Evergreen tree	Ferrar *et al.*, 1989
*Eucalyptus pauciflora* (PS)	Evergreen tree	Ferrar *et al.*, 1989
*Eucalyptus pauciflora* (WP)	Evergreen tree	Ferrar *et al.*, 1989
*Eucryphia lucida* F700	Evergreen tree	Read and Busby, 1990
*Gentiana straminea*	C_3_ herb	Shen *et al.*, 2009, 2013
*Geraea canescens*	C_3_ herb	Toft and Pearcy, 1982
*Geum rivale*	C_3_ herb	Graves and Taylor, 1988
*Geum urbanum*	C_3_ herb	Graves and Taylor, 1988
*Glycine max*	Evergreen tree	Rosenthal *et al.*, 2014
*Helianthus mollis*	C_3_ herb	Zhou *et al.*, 2007
*Lagarostrobos franklinii* P80	Evergreen tree	Read and Busby, 1990
*Larix decidua*	Deciduous tree	Tranquillini *et al.*, 1986
*Liquidambar styraciflua*	Deciduous tree	Dillaway and Kruger, 2010
*Lupinus arizonicus*	C_3_ herb	Forseth and Ehleringer, 1982
*Malvastrum rotundifolium*	C_3_ herb	Forseth and Ehleringer, 1982
*Mucuna pruriens*	C_3_ herb	Monson *et al.*, 1992
*Nothofagus cunninghamii* F700	Evergreen tree	Read and Busby, 1990
*Nothofagus cunninghamii* F980	Evergreen tree	Read and Busby, 1990
*Nothofagus cunninghamii* P80	Evergreen tree	Read and Busby, 1990
*Nothofagus gunnii* F980	Evergreen tree	Read and Busby, 1990
*Oryza sativa*	C_3_ herb	Nagai and Makino, 2009
*Phaseolus vulgaris*	C_3_ herb	Cowling and Sage, 1998
*Phyllocladus aspleniifolius* F700	Evergreen tree	Read and Busby, 1990
*Picea abies*	Evergreen tree	Kroner and Way, 2016
*Picea koraiensis*	Evergreen tree	Zhang *et al.*, 2015
*Picea likiangensis* var. *linzhiensis*	Evergreen tree	Zhang *et al.*, 2015
*Picea likiangensis* var. *rubescens*	Evergreen tree	Zhang *et al.*, 2015
*Picea mariana*	Evergreen tree	Way and Sage, 2008*a* ,b
*Picea meyeri*	Evergreen tree	Zhang *et al.*, 2015
*Plantago asiatica* (Sendai)	C_3_ herb	Ishikawa *et al.*, 2007
*Plantago asiatica* (Shimada)	C_3_ herb	Ishikawa *et al.*, 2007
*Plantago asiatica* (Tomakomai)	C_3_ herb	Ishikawa *et al.*, 2007
*Populus balsamifera* (cool)	Deciduous	Silim *et al.*, 2010
*Populus balsamifera* (warm)	Deciduous	Silim *et al.*, 2010
*Populus deltoides*	Deciduous	Dillaway and Kruger, 2010
*Populus tremula* × *Populus tremuloides*	Evergreen	Rasulov *et al.*, 2015
*Populus tremuloides*	Deciduous	Dillaway and Kruger, 2010
*Quercus rubra*	Deciduous	Gunderson *et al.*, 2009
*Schima superba*	Evergreen	Sheu and Lin, 1999
*Simmondsia chinensis*	Evergreen	Wardlaw *et al.*, 1983
*Sorghastrum nutans*	C_4_	Zhou *et al.*, 2007
*Spinacia olearacea*	C_3_ herb	Yamori *et al.*, 2006
*Stipa krylovii*	C_3_ herb	Chi *et al.*, 2013
*Triticum aestivum*	C_3_ herb	Nagai and Makino, 2009
*Triticum aestivum*	C_3_ herb	Yamasaki *et al.*, 2002

## Perspectives

Although Slot and Winter (2017) provide critical data on how carbon fluxes in tropical species acclimate to warming, there is a pressing need to move beyond gas exchange measurements in these types of studies. Many papers on thermal acclimation measure traits such as leaf nitrogen concentrations and specific leaf area, but future studies should delve more deeply into the biochemical and physiological mechanisms underlying photosynthetic (and respiratory) acclimation. Recent studies in tropical tree species have highlighted the importance of within-leaf N allocation as a strong determinant of variation in photosynthetic capacity (Coste *et al.*, 2005; Dusenge *et al.*, 2015). Specifically, Scafaro *et al.* (2016) demonstrated that accounting for changes in N allocation to the CO_2_-fixing enzyme Rubisco in response to growth temperature explained the measured variation in photosynthetic capacity in a range of temperate and tropical species. Shifts in N allocation between the Calvin cycle and electron transport may represent a major theme for thermal acclimation of carbon gain (Hikosaka *et al.*, 2006), but we still lack a predictive model of photosynthetic acclimation to temperature that could explain the variation we see between plant functional types (as described in Yamori *et al.*, 2014, and Way and Yamori, 2014). While this is not a problem unique to tropical systems, building such a model will require a much more extensive understanding of how changes in temperature affect photosynthesis in a broad range of species and ecosystems. This represents a significant challenge, but it would be an important step forward for predicting future carbon fluxes in vegetation.
